# Dyspeptic Origin of Visual Troubles

**Published:** 1887-08

**Authors:** 


### DYSPEPTIC ORIGIN OF VISUAL TROUBLES.

IN the opthalmological congress, M. Grandelement, of Lyons, said: "It is often observed that there are intermittent or remittent visual troubles which one can explain neither by the anomalies of refraction, nor by lesions of the media, or of the deep membranes of the eye. It is common in such cases to attribute these in a stupid way to the nerves. But if we scrutinize with attention the entire organism, we shall always find in such cases a defective condition of the digestive functions;—a dyspepsia, perhaps more or less chronic, and sometimes also a fault of nutrition (excess of phosphates, glycosuria, etc.). These functional affections of vision, this kind of neuropathic phenomenon, constitutes one of those numerous sympathetic neuroses caused by a faulty assimilation of the food, and the formation of toxic alkaloids or ptomaines, which result in the most various distant troubles. The visual defects observed most frequently consist in a diffused pain of the globe, radiating toward the temples, the forehead, and even to the scalp. There is hyperæsthesia of the retina, with photophobia and photopsia, more or less severe, following the limited exercise of accommodation. Other patients complain of scotomæ, hemianopsia, diplopia, and especially of *mouches volantes*. All of these ocular neuropathies may be cured, or their trouble, at least, greatly ameliorated, by careful attention to dietetics and hygiene, and by the employment of saline laxatives and alkalies, in moderate doses and for a long time. In the most severe cases, flushing the stomach, rest in bed, or the use of a hypogastric girdle to sustain the dilated stomach and intestines, will produce the best results."—*Le Concours Médical, May 28, 1887.*

### RETENTION OF URINE.

DR. John Ashhurst, Jr., Professor of Clinical Surgery in the University of Pennsylvania, in a clinical, lecture, says:

I shall take this opportunity of making a few remarks on the subject of retention of urine. Retention must, in the first place, be distinguished from suppression. The latter is a much graver condition, and depends upon some fault in the kidney, either from organic disease or from sympathy with some other portion of the body. As a result the urine is not secreted, and the waste matters remaining in the blood soon give rise to symptoms of uræmia. In retention the urine is secreted but is not passed, and accumulates in the bladder. If there be free secreetd of urine, and only retention, the patient may live almost indefinitely, particularly when the retention is of a chronic character, such as we meet with in the case of old persons with enlargement of the prostate. In these cases, retention more or less complete may last for many months. Some years ago a patient presented himself at this hospital, with the statement that his urine was running away all the time. He had been under treatment for paralysis of the bladder. On examination, the bladder was found to be greatly distended, the vesical tumor reaching to the umbilicus. There was a certain amount of dribbling, but the bladder had remained full of urine for at least a year. The whole trouble was due to enlargement of the prostate.

Retention of urine may be due to several conditions. It may be the result of spasm. If a person is obliged to hold his water for an unusual length of time, it not unfrequently happens that when he attempts to empty the bladder he finds that he is unable to do so on account of spasm at the neck of the organ. Retention may also depend upon certain general conditions of the system : thus, in typhoid fever retention of urine is a common occurrence. There it is due to the fact that the nervous system, being under the influence of the poison of the disease, fails to recognize the necessity of emptying the bladder. The bladder, under these circumstances, may become much distended and great harm result.

Among the conditions that more commonly come under the observation of the surgeon is what has been termed spasmodic stricture, which is a condition very similar to the spasm at the neck of the bladder already referred to. A person becomes chilled from exposure to cold, and when he attempts to pass water finds that he is unable to do so on account of spasm. There is another condition which is termed congestive retention. Under such circumstances there is probably always some organic change, some stricture, which under ordinary circumstances gives no annoyance ; but, as a result of some

